# Formyl peptide receptor 1 up-regulation and formyl peptide receptor 2/3 down-regulation of blood immune cells along with defective lipoxin A4/resolvin D1 production in obstructive sleep apnea patients

**DOI:** 10.1371/journal.pone.0216607

**Published:** 2019-05-22

**Authors:** Yung-Che Chen, Mao-Chang Su, Chien-Hung Chin, I-Chun Lin, Po-Yuan Hsu, Chia-Wei Liou, Kuo-Tung Huang, Ting-Ya Wang, Yong-Yong Lin, Yi-Xin Zheng, Chang-Chun Hsiao, Meng-Chih Lin

**Affiliations:** 1 Division of Pulmonary and Critical Care Medicine, Department of Medicine, Kaohsiung Chang Gung Memorial Hospital and Chang Gung University College of Medicine, Kaohsiung, Taiwan; 2 Sleep Center, Kaohsiung Chang Gung Memorial Hospital and Chang Gung University College of Medicine, Kaohsiung, Taiwan; 3 Department of medicine, Chang Gung University, Taoyuan, Taiwan; 4 Chang Gung University of Science and Technology, Chia-yi, Taiwan; 5 Department of pediatrics, Kaohsiung Chang Gung Memorial Hospital and Chang Gung University College of Medicine, Kaohsiung, Taiwan; 6 Department of medical research, Kaohsiung Chang Gung Memorial Hospital and Chang Gung University College of Medicine, Kaohsiung, Taiwan; 7 Department of neurology, Kaohsiung Chang Gung Memorial Hospital and Chang Gung University College of Medicine, Kaohsiung, Taiwan; 8 Graduate institute of clinical medical sciences, College of Medicine, Chang Gung University, Taoyuan, Taiwan; National Yang-Ming University, TAIWAN

## Abstract

**Background:**

This study aims to investigate the role of FPR 1/2/3 expressions in patients with obstructive sleep apnea (OSA).

**Method:**

We made cross-sectional comparisons of FPR1/2/3 expressions of blood neutrophil, M1/M2a monocyte, and natural killer (NK) cell between 16 healthy subjects (HS), 16 primary snoring (PS) subjects, 46 treatment-naive OSA patients, and 18 severe OSA patients under long-term continuous positive airway pressure treatment (severe OSA on CPAP).

**Results:**

FPR1 expressions on neutrophil were increased in treatment-naive OSA and severe OSA on CPAP groups versus either HS or PS. FPR2 expressions on neutrophil were decreased in treatment-naive OSA versus HS, and returned to normal in severe OSA on CPAP group. FPR1/FPR2 expression ratio on neutrophil was increased in treatment-naive OSA versus either HS or PS. Serum lipoxin A4, resolvin D1 levels, and FPR3 expressions of M1, M2a and NK cells were all decreased in treatment-naive OSA versus HS. OSA patients with hypertension had decreased FPR2 expressions on neutrophil and FPR3 expressions of NK cell. FPR1 expression, FPR1/FPR2 expression ratio on neutrophil, and FPR3 expression of M1 cell were all reversed after > 6-month CPAP treatment in 9 selected patients. In vitro intermittent hypoxia with re-oxygenation treatment in THP-1 cells resulted in increased FPR1/FPR2 expression ratio of M1 cells, and increased FPR1/FPR3 expression ratio of M2a cells.

**Conclusions:**

FPR1 over-expression and insufficiency of FPR2 and FPR3 in association with defective lipoxin A4 and resolving D1 production were associated with disease severity of OSA and its adverse consequences.

## Introduction

Obstructive sleep apnea (OSA) is a clinical condition characterized by recurrent episodes of complete or partial obstruction of the upper airway, leading to sleep fragmentation and chronic intermittent hypoxia with re-oxygenation (IHR) during sleep. In parallel with ischemia-reperfusion injury, chronic IHR in OSA leads to sympathetic nervous system activation and oxidative stress with a resultant systemic inflammatory cascade and subsequent endothelial dysfunction, which may contribute to increased risk of cardiovascular disease, hypertension, stroke, and all-cause mortality[1, 2]. Apnea hypopnea index (AHI) is independently associated with impaired endothelial function[3]. Although the administration of continuous positive airway pressure (CPAP) can partly reverse changes associated with systemic inflammation, endothelial dysfunction, and systemic blood pressure in OSA patients[4, 5], it may not prevent cardiovascular events in patients with OSA and established cardiovascular disease[6].

Formyl peptide receptors (FPRs) belong to the seven-transmembrane G protein-coupled receptor superfamily and chemoattractant receptors, which encompass three subtypes, including FPR1, ALX (Aspirin-triggered Lipoxin)/FPR2 (or Lipoxin A4 (LXA4) receptor), and FPR3. FPR1 and FPR2 are evenly distributed on the cell surface, while FPR3 is localized within the cytoplasm [7]. FPR1 is associated with pro-inflammatory responses, such as cytokine production, and superoxide production, while FPR2 is an unconventional receptor because it can convey contrasting biological signals, depending on the ligands[8, 9]. LXA4, resolving D1 (RvD1), and annexin A1 (ANXA1) ligations result in FPR2/FPR2 homodimerization, which leads to anti-inflammatory responses, such as interleukin-10 generation, via activation of p38/MAPK/APK/Hsp27 pathway [10]. In contrast, serum amyloid protein A (SAA) and cathelicidin (LL-37) ligations result in FPR2/FPR1 heterodimerization, which leads to the induction of pro-inflammatory responses via JNK pathway signaling [10, 11]. FPR3 is supposed to be a decoy receptor, which does not transduce signal, but undergoes rapid constitutive recycling to bind extracellular ligand and internalize it for degradation[7].

FPR1 genetic variant is associated with higher 5-year increase of blood pressure levels in healthy individuals aged less than 45 years[12]. Recent studies have shown that FPR1 modulates endothelial cell functions by NADPH oxidase-dependent VEGF receptor 2 transactivation and inducing reactive oxygen species generation, while FPR 2 is involved in cardiovascular repair after myocardial infarction and stroke through mobilization of circulating angiogenic cells and reducing leukocyte-endothelial interactions. On the other hand, ANXA1 and RvD1 have a protective effect against ischemia reperfusion induced-acute lung injury and hypoxia-induced inflammation, respectively [13–18].

We hypothesized that FPR1/2/3 expressions of blood innate immune cells, including neutrophil, monocyte, and natural killer (NK) cell, and serum protein levels of five FPR ligands, including LXA4, RvD1, ANXA1, SAA, and LL-37, may be associated with disease severity, clinical phenotypes, or endothelial dysfunction of OSA syndrome. Thus, we conducted this prospective cohort study to investigate the relationship between FPR1/2/3/five PFR ligands protein expressions and sleep parameters in 80 patients with sleep disordered breathing and 16 healthy subjects (HS) without snoring history and OSA symptoms. These biomarkers were examined again after more than 6-month home CPAP treatment in selected OSA patients. Additionally, the relation between endothelial function, as determined by ultrasound assessment of flow mediated dilatation at brachial artery, and FPR1/2/3 expressions were evaluated in 6 treatment-naïve severe OSA patients and 6 matched HS. Furthermore, the effects of in vitro IHR on FPR1/2/3 expressions of human monocytic cell lines were determined.

## Methods

### Subjects

**Fig 1:**
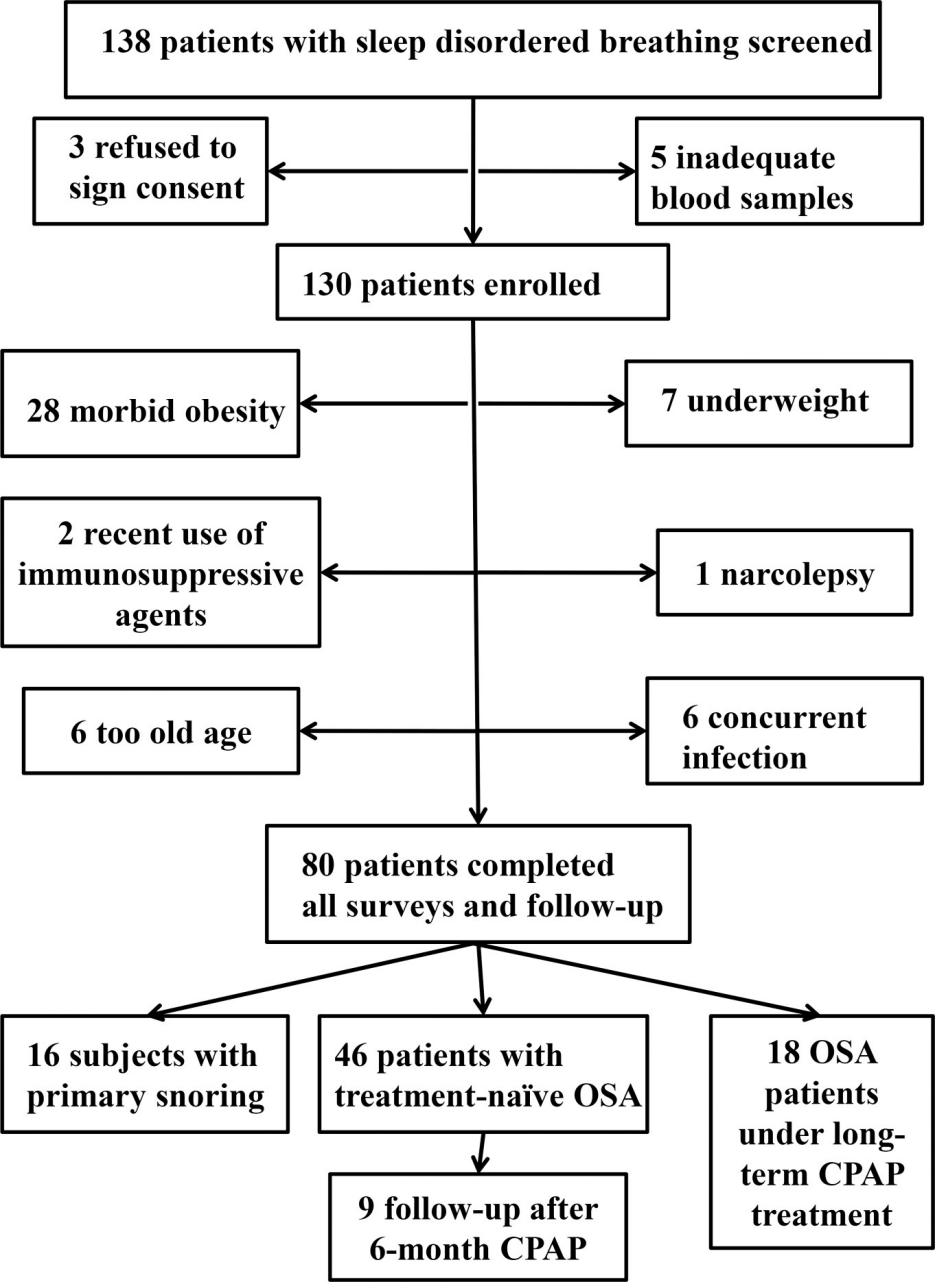
Flow diagram shows the process of the patients’ selection and enrollment.

The study was approved by the Institutional Review Board of Chang Gung Memorial Hospital, Taiwan (certificate number: 102-5855B). Written informed consent was obtained from all study participants, including SDB subjects and HS. All participating subjects who presented with loud snoring were recruited from the sleep center and pulmonary clinics of Kaohsiung Chang Gung Memorial Hospital during the period from August 2014 through May 2017. One hundred thirty eight subjects presenting with loud snoring underwent full-night PSG exam and were screened. Exclusion criteria included narcolepsy, ongoing infections, autoimmune disease, recent use of immunosuppressive agent, morbid obesity (defined as body mass index, BMI, >34 kg/m2), elderly (>65 years old), and underweight (BMI<21 kg/m2). A total of 80 subjects were consecutively included for study (**Fig 1**). Among them, 16 subjects with primary snoring (PS, apnea hypopnea index, AHI <5.0), 46 treatment-naïve patients with OSA (AHI≧5.0), and 18 severe OSA on long-term CPAP treatment (AHI≥30.0 at diagnosis and CPAP use ≥ 4 hour/night for ≥one year) patients were enrolled for final analysis. Very severe OSA was defined as AHI≧50.0, since patients with severe OSA exhibited higher mortality compared with those with mild and moderate OSA, and those with AHI≧50 had even higher hazard ratio for mortality compared with those with severe OSA[1, 19]. Additional 16 HS without habitual snoring and related OSA symptoms, which were reported by the subjects and their bed partners, were enrolled from the health examination center of Kaohsiung Chang Gung Memorial Hospital. Associated co-morbidities and adverse consequences of OSA, including hypertension (defined as baseline blood pressure>140/90 mmHg or prescription of anti-hypertension medicines), cardiac disease (including history of ischemic heart disease, dysrhythmia, and heart failure), diabetes mellitus, cerebral vascular accident, and excessive daytime sleepiness (defined as Epworth Sleepiness Scale [20] >10), were recorded.

### Polysomnography and CPAP titration study

All the SDB patients underwent overnight polysomnohraphy examination at the sleep laboratory of Kaohsiung Chang Gung Memorial Hospital. Polysomnography measurements were conducted by using Sandman SD32+TM Digital Amplifier (Embla, Colorado, U.S.A.). Three trained technicians performed sleep stage scoring according to standard criteria [21]. The percentage of sleep recording time with oxy-hemoglobin saturation <90% (%time <90% SaO2), minimum SaO2, mean SaO2, and the number of dips >4% of basal SaO2%//hour (oxygen de-saturation index, ODI) were recorded, indicating nocturnal hypoxia statuses. Snoring sampling was performed at 10 to 50 Hz, and all sounds of more than 50% above baseline amplitude lasting for 0.5–5 sec were recorded by Piezo crystal snore sensor (SleepSense, Scientific Laboratory Products, Elgin, USA). OSA patients would undergo manual CPAP titration study (GoodKnight 420E, Nellcor Puritan Bennett, California, U.S.A.) at the sleep laboratory to get an optimal pressure before starting either fixed or auto-adjusted positive airway pressure machine treatment at home.

### Measurements of cell surface FPR1/2 and intracellular FPR3 protein expressions of peripheral blood CD14^+^CD209^-^ M1 monocyte, CD14^+^CD209^+^ M2a monocyte, CD16^+^neutrophil, CD3^+^CD56^+^ NKT cell, and CD3^-^CD56^+^ NK cell by flowcytometry

Twenty ml venous blood was withdrawn from all study participants at 07:30–08:30AM after overnight fast and sleep. Additional 20 ml venous blood was withdrawn at around 07:30–08:30AM from 9 OSA patients after > 6-month CPAP treatment. A panel of monoclonal antibodies consisting of isotype control (mouse IgG conjugated to fluorescein isothiocyanate (FITC) and phycoerythrin (PE)), monocyte marker (CD14-PE-CyTM7, BD Pharmingen), neutrophil marker (CD16-PC5, Beckman Coulter), NK cell marker (CD3-PE-CyTM5, PE-Cy7), and T cell marker (CD3- PE-CyTM5, BD Pharmingen) were used to identify specific immune cells. To measure cell surface FPR1/2 expressions on blood neutrophils, and mononuclear cells separately, FPR1-CFS (R&D) and FPR2-PE (R&D) in combination with immune cell markers were used. For intracellular staining of FPR3, cells were stained with immune cell markers and incubated with Cytofix/CytopermTM, followed by incubation with anti-FPR3 PE (Santa Cruz) or isotype control mAb. After erythrocytes were lysed with OptiLyse C Lysis solution (Beckman Coulter; France), acquisition with a dual staining protocol was performed on a CytomicsTM FC500 (Beckman Coulter; USA). Immune cells were gated in a side scatter versus frontal scatter plot with a collection of 5×10^5^ events each time. These were further analyzed for FPR1/2/3 expressions of various immune cells in the FL1 and FL2 channels, respectively, by using CXP Analysis software. Mean fluorescence intensity (MFI) with correction for background fluorescence, and percentage of FPR (+) cells were recorded. Representative plots and histograms showing cell surface FPR1/2 expression on CD16^+^ neutrophil and intracellular FPR3 expression of CD14^+^ monocyte are presented in **S1 Fig**.

### Measurements of endothelial function by flow-mediated dilatation

The diameter of the brachial artery was measured in triplicate at baseline and during reactive hyperemia, using a high-resolution ultrasound sphygmomanometer with a 7-10-MHz linear array transducer (Aloca Ultrasound, USA). Longitudinal images of the brachial artery were obtained proximal to the antecubital fossa with the transducer secured by the probe holder. Transmit focus zones were set approximately to the depths of the anterior and the posterior vessel walls. Images were magnified, and depth and gain settings were used to optimize the image of the vessel wall, in particular the media-adventitia interface.

Ultrasound of the brachial artery was performed by an independent cardiologist in a quiet and dimly darken room at the temperature of 21–23°C. All patients were allowed to rest for 10 min before the first scan. After baseline data was recorded for 1 minute, increased flow was then induced by inflation of a pneumatic tourniquet placed around the wrist to a pressure of 250 mm Hg for 5 min. A second scan was obtained 45–60 s after cuff deflation, and data was recorded for 10 minutes. Maximum vessel diameters (VD) after reactive hyperemia was compared to the diameters at baseline, and expressed as a percentage to the average lumen diameter at baseline: Flow mediated dilatation (%) = [(VD reactive hyperemia–VD at baseline) × 100]/VD baseline. Arterial blood flow was measured as flow velocity multiplied by the cross-sectional area (π× r ^2^) [22].

### Measurements of serum levels of SAA, LL-37, LXA4, ANXA1 and RvD1

Serum SAA, LL-37, LXA4, ANXA1 and RvD1 levels were measured by a commercial enzyme-linked immunosorbent assay (ELISA) method (INVITROGEN, USA; HYCULTBIOTECH, USA; Cusabio, China; ASSAYPRO, USA; CAYMAN, USA) according the manufacture’s manual.

### In vitro human monocyte cell line culture under IHR stimuli

The human monocytic leukemia cell line THP-1 cells obtained from ATCC (1×10^6^ cells/ml) were re-suspended in a 5 cm culture dish containing 5 ml RPMI 1640 medium, and then exposed to normoxia (NOX), persistent hypoxia (PH) or IHR in a custom-designed, incubation chambers which are attached to an external O2-CO2-N2 hand-driven controller as previously described[23]. Air-phase set point consisted of a 15-min hypoxic period (0% O2 and 5% CO2), followed by 15 min of re-oxygenation (21% O2 and 5% CO2) for IHR, or continuous hypoxia (0% O2 and 5% CO2) for PH, 7 h each day for 2 days. Cells maintained in NOX condition for the same durations were used as control. The amount of viable THP-1 cells under the stimuli with IHR, PH, or NOX for 48 h was determined via optical density measurement of WST-1 reagent (Roche, Mannheim, Germany) diluted at 1:10 using a microplate reader at 450 nm, with 600 nm as a reference wavelength.FPR1/2/3 protein expression levels of the THP-1 cells were measured by flowcytometry method. All the experiments were performed independently for 6 times.

### Statistical analysis

Data were expressed as the mean±standard deviation. Mann–Whitney U test was used for comparing continuous values of two experimental groups, and Wilcoxon singed rank test was used for comparing continuous values before and after treatment. Kruskal-Wallis test followed by post hoc analysis was used for comparing median values of more than two experimental groups. Chi-square test was used to analyze categorical variables. Spearman's rank correlation test was used to determine the relationship between continuous variables. Stepwise multiple linear regression analysis was used to minimize the effects of confounding factors in the subgroup analyses with all potential co-variables, including age, sex, BMI, smoking history, alcoholism history, and co-morbidities, entered in a single step, and to get adjusted p values. A p value of less than 0.05 is considered statistically significant. A statistical software package (SPSS, version 20.0, Chicago, IL) was used for all analyses.

## Results

### Demographic data

A total of 16 HS and 80 patients with sleep disordered breathing were classified into four groups according to history of habitual snoring, AHI and history of long-term CPAP treatment. Demographic, polysomnography, and blood chemistry data of all the study participants are listed in **Table 1**. There were not any significant differences between four groups in terms of age, sex, smoking history, co-morbidity, and blood biochemical profiles. BMI was significantly higher in the treatment-naive OSA (p = 0.033) and severe OSA on CPAP groups (p = 0.031) than that in the HS group. Polysomnography parameters and Epworth Sleepiness Scale differed significantly between four groups Post hoc comparisons revealed that subjects in the severe OSA on CPAP group had higher Epworth Sleepiness Scale than subjects in the PS and treatment-naive OSA groups, but ESS did not differ between the PS and treatment-naive OSA groups. Eleven patients in the severe OSA on CPAP group had undergone manual CPAP titration study before regular CPAP treatment at home, showing significant improvement in AHI (0.5±1 vs. 60.8±14.1 events/hr, p = 0.003) and snoring index (13.3±26.9 vs.416.6±244.8 counts/hr, p = 0.005) at their optimal titration pressure (5–10 cmH2O).

**Table 1:** Demographic, biochemistry, and sleep data of all the 96 study participants.

	HS (n = 16)	PS (n = 16)	Treatment-naïve OSA (n = 46)	Severe OSA on CPAP (n = 18)	p value
Age, years	55.1±12.1	46.4±8.9	48.4±12.8	51.7±10.8	0.179
Male Sex, n (%)	15 (93.8)	12 (75)	38 (82.6)	16 (88.9)	0.42
BMI, kg/m^2^	24.9±3.4	25.9±3.8	27.0±2.8	27.4±4.3*	0.048
AHI, events/hour	NA	3.9±3.4	26.0±2.8#	62.8±14.5#@	<0.001
ODI, events/hour	NA	1.2±1.2	28.3±4.2#	53.3±19.8#@	<0.001
Mean SaO2, %	NA	96.8±1.3	94.5±3.5#	93.0±2.1#	<0.001
Minimum SaO2, %	NA	91.1±3.4	75.9±13.1#	71.3±9.6#	<0.001
Snoring index, counts/hour	N A	139.9±178.6	326.3±257.7#	328.5±274.5#	0.043
Epworth Sleepiness Scale	NA	7.8±4.6	9.8±3.9	15.2±6#@	0.001
Excessive daytime sleepiness, n (%)	NA	4 (25)	18 (39.1)	16 (88.9)	0.001
Current smoking, n (%)	0 (0)	4 (25)	7 (15.2)	2 (14.3)	0.263
Cholesterol, mg/dl	185.7±43.6	192.9±19.5	178.6±40.8	183.9±31.2	0.812
Triglycerides, mg/dl	114.9±55.8	140.6±77.1	135.5±72.8	196.4±138.3	0.07
White blood cell counts, μL^-1^	5725±2103	7392±4720	6797±1743	7858±2461	0.167
Neutrophil, %	56.1±9.5	60.2±10.3	56.3±8.4	59.4±6.2	0.134
Lymphocyte, %	33.9±7.9	30.6±9.5	34.6±8.0	32.2±5.9	0.134
Monocyte, %	6.1±1.9	5.5±0.9	5.4±1.4	5.2±1.5	0.689
Eosinophil, %	3.4±3.6	3.3±2	3.0±2.1	2.7±1.5	0.874
Basophil, %	0.4±0.3	0.4±0.2	0.4±0.2	0.3±0.1	0.677
Hemoglobin,g/dL	14.7±0.9	14.2±1.3	15.1±1.6	14.5±0.9	0.206
Red blood cell counts, 10^6^/μL	4.9±0.3	4.8±0.5	5.0±0.5	5.4±0.9	0.487
Platelet counts, 10^3^/μL	198±36	210±37	223±45	224±57	0.789
Hypertension, n (%)	2 (12.5)	4 (25)	10 (21.7)	9 (50)*	0.048
Diabetes mellitus, n (%)	1 (6.3)	1 (6.3)	6 (13)	2 (11.1)	0.489
Heart disease, n (%)	0 (0)	0 (0)	3 (5.9)	3 (6.5)	0.465
Stroke, n (%)	0	1 (5.9)	1 (2.4)	0	0.224
CKD, n (%)	0	0	0	0	1

PS = primary snoring; HS = healthy subjects; BMI = body mass index; AHI = apnea hypopnea index; ODI = oxygen desaturation index; SaO2 = arterial oxyhemoglobin saturation; CKD = chronic kidney disease (defined as an estimated glomerular filtration rate of less than 60 ml/min/1.73m^2^ on at least 2 occasions 90 days apart)

*p<0.05 compared with HS group by Kruskal-Wallis test or Chi-square test, where appropriate

#p<0.05 compared with PS group by Kruskal-Wallis test or Chi-square test, where appropriate

@p<0.05 compared between treatment-naïve OSA and severe OSA on CPAP groups by Kruskal-Wallis test or Chi-square test, where appropriate

### Increased FPR1 protein expressions and decreased FPR2 protein expression on blood CD16^+^ neutrophil in treatment naive OSA patients

**Fig 2:**
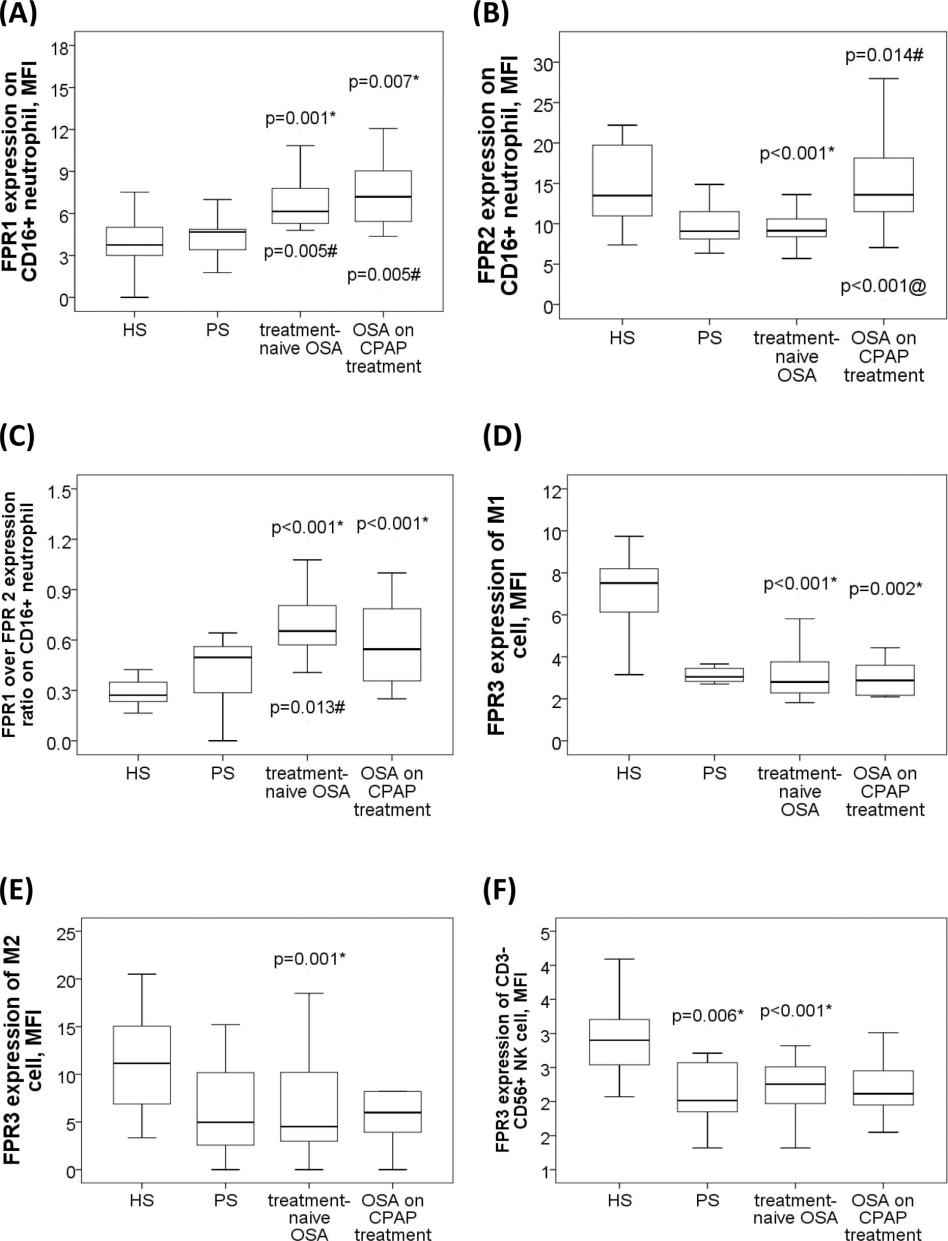
Formyl peptide receptor (FPR) 1/2/3 expressions of blood innate immune cells in patients with sleep disordered breathing and healthy subject without snoring. **(A)** Increased FPR1 expression on neutrophil in patients with treatment-naive obstructive sleep apnea (OSA), and severe OSA under long-term CPAP treatment groups as compared with that in subjects with primary snoring (PS) and healthy subjects (HS). (B) Decreased FPR2 expression on neutrophil in treatment-naïve OSA group as compared with that in HS, and severe OSA on CPAP groups. (C) Increased FPR1/FPR2 expression ratio in treatment-naïve OSA and severe OSA on CPAP groups as compared with that in HS group. (D) Decreased FPR3 expression of M1 monocyte in treatment-naïve OSA and severe OSA on CPAP groups as compared with that in HS group. (E) Decreased FPR3 expression of M2a monocyte in treatment-naïve OSA versus HS group. (F) Decreased FPR3 expression of NK cell in PS, and treatment-naïve OSA, groups as compared with that in HS group. The box plots show the 25th, 50th, 75th percentiles, maximum, and minimum. *P<0.05 for comparisons between HS and another groups by Kruskal-Wallis test followed by post-hoc corrections and linear regression adjustments #p<0.05 for comparisons between PS and another groups by Kruskal-Wallis test followed by post-hoc corrections and linear regression adjustments @p<0.05 for comparisons between treatment-naïve OSA and severe OSA on CPAP groups by Kruskal-Wallis test followed by post-hoc corrections and linear regression adjustments.

There were significant between-group differences in FPR1 and FPR2 expression MFI on neutrophil (both p values <0.05). Post hoc comparisons with corrections for multiple comparisons and adjustment for confounding factors revealed that cell surface FPR1 protein expressions on neutrophil were significantly increased in the treatment-naive OSA (7.24±3.2 MFI; p = 0.009/adjusted p = 0.001 vs. HS; p = 0.009/adjusted p = 0.005 vs. PS) and severe OSA on CPAP (8.72±5.07 MFI; p = 0.001/adjusted p = 0.007 vs. HS; p = 0.001/adjusted p = 0.005 vs. PS) groups as compared with that in the HS (4.12±0.96 MFI) and PS (4.11±1.66 MFI) groups, respectively (**Fig 2A**). Cell surface FPR2 protein expressions on neutrophil were decreased in the treatment-naive OSA (9.71±3.32 MFI, p = 0.001/adjusted p<0.001) group as compared with that in the HS (14.93±5.11) group, and increased in the severe OSA on CPAP (15.89±6.78 MFI) group as compared with that in the treatment-naive OSA (p<0.001/adjusted p<0.001) group and that in the PS (p = 0.001/adjusted p = 0.014) group, respectively (**Fig 2B**).

### Increased FPR1/FPR2 expression ratio on blood CD16^+^ neutrophil in treatment naive OSA patients

**Fig 3:**
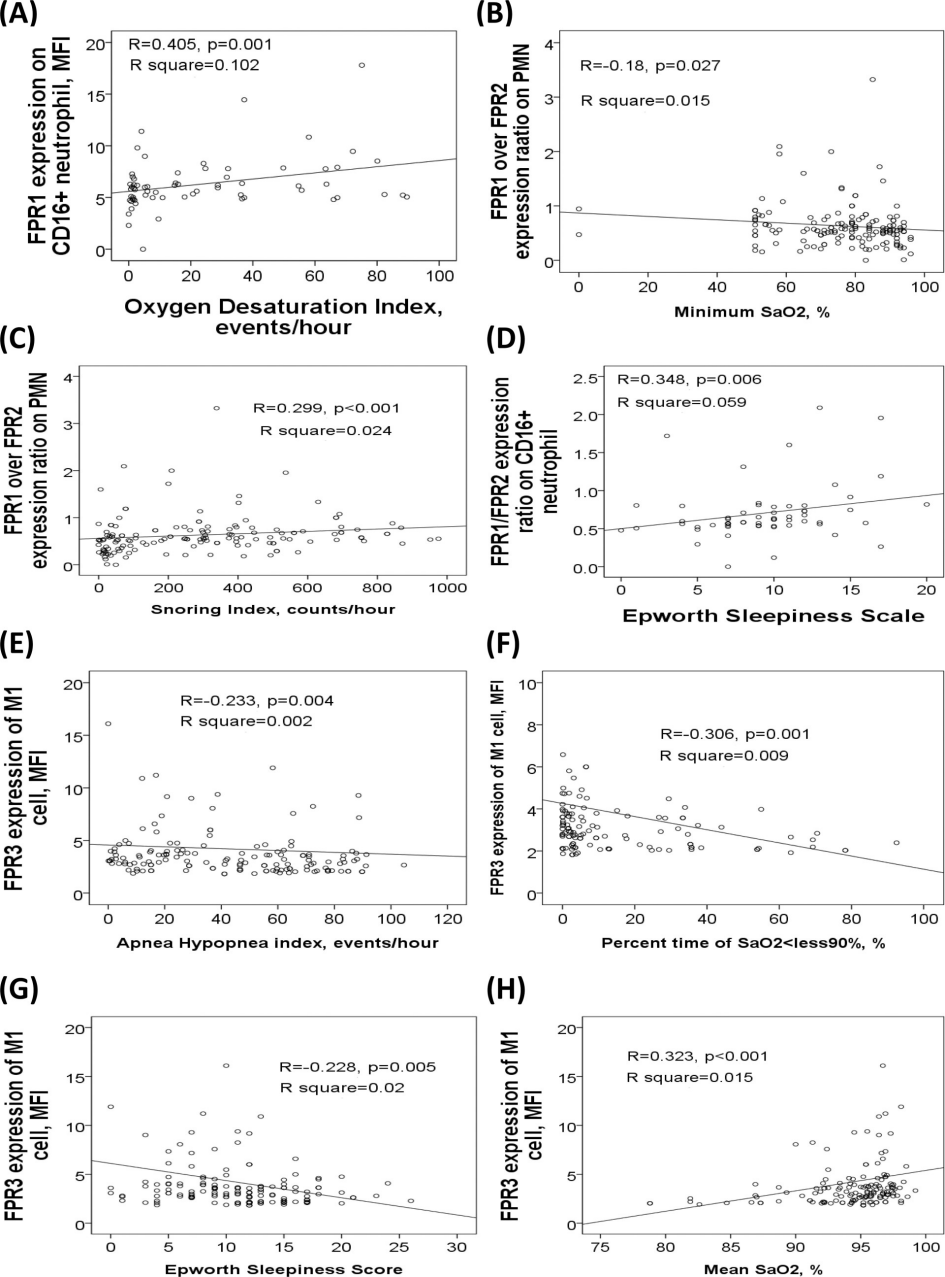
Correlations of FPR expressions of blood immune cells with sleep parameters. **(A)** FPR1 expression on neutrophil was positively correlated with ODI. FPR1/FPR2 expression ratio was (B) negatively correlated with minimum SaO2, and positively correlated with (C) snoring index and (D) Epworth Sleepiness Scale. FPR3 expression of M1 monocyte was negatively correlated with (E) apnea hypopnea index, (F) percent time of SaO2<90%, and (G) Epworth Sleepiness Scale, and (H) positively correlated with mean SaO2.

FPR1/FPR2 expression ratio on neutrophil was significantly increased in the treatment-naive OSA (0.73±0.42) group as compared with that in the HS group (0.28±0.1 MFI; p = 0.001/adjusted p = 0.001) and that in the PS group (0.41±0.18 MFI; p<0.001/adjusted p = 0.013), and also increased in the severe OSA on CPAP group (0.56±0.23 MFI, p<0.001/adjusted p = 0.023) as compared with that in the HS group (**Fig 2C**). FPR1 expression on neutrophil was positively correlated with snoring index (r = 0.171, p = 0.036) and ODI (r = 0.405, p = 0.001, **Fig 3A**). FPR1/FPR2 expression ratio on neutrophil was negatively correlated with minimum SaO2 value (r = -0.18, p = 0.027, **Fig 3B**), and positively correlated with both snoring index (r = 0.299, p<0.001, **Fig 3C**) and ESS values (r = 0.348, p = 0.006, **Fig 3D**). Cell surface FPR1 and FPR2 protein expressions on CD14^+^CD209^-^ M1 monocyte, CD14^+^CD209^+^ M2a monocyte, CD3^+^CD56^+^ NK T cell, or CD3^-^CD56^+^ NK cell were not different among the 4 groups.

### Decreased intracellular FPR3 expressions of M1 monocyte and M2a monocyte in treatment-naïve OSA patients

There were significant between-group differences in FPR3 expression MFI of M1 monocyte, M2a monocyte and NK cell (all p values <0.01). Intracellular FPR3 expression of M1 monocyte was significantly decreased in treatment-naive OSA (3.29±1.54, MFI, p<0.001/adjusted p<0.001) and severe OSA on CPAP (3.17±1.71 MFI, P<0.001/adjusted p = 0.002) groups as compared with that in HS (7.54±2.75 MFI) group (**Fig 2D**), while FPR3 expression of M2a monocyte was significantly decreased in the treatment-naïve OSA group as compared with that in the HS group (6.21±5.14 versus 11.38±5.12; p = 0.016/adjusted p = 0.001; **Fig 2E**). Intracellular FPR3 expression of M1 monocyte was negatively correlated with AHI (r = -0.233, p = 0.004, **Fig 3E**), %time <90% SaO2 (r = -0.306, p = 0.001, **Fig 3F**), ODI (r = -0.272, p = 0.001), and ESS (r = -0.228, p = 0.005, **Fig 3G**), and positively correlated with both mean SaO2 (r = 0.323, p<0.001, **Fig 3H**) and minimum SaO2 (r = 0.32, p<0.001).

### Decreased intracellular FPR3 expression of NK cell in treatment-naïve OSA patients

Intracellular FPR3 expression of NK cell was significantly decreased in PS (2.1±0.4 MFI, p<0.001/adjusted p<0.001), treatment-naive OSA (2.28±0.43 MFI, P<0.001/adjusted p = 0.006), and severe OSA on CPAP (2.15±0.39 MFI, p<0.001/adjusted p<0.001) groups as compared with that in HS (2.94±0.58 MFI) group (**Fig 2F**). Intrcellular FPR3 expressions of neutrophil and NK T cell were not different among the 4 groups.

### Decreased FPR2 expression on neutrophil and decreased FPR3 expression of NK cell in sleep disordered breathing patients with hypertension

Subgroup analysis in treatment-naïve sleep disordered breathingpatients, including PS and treatment-naive OSA groups, showed that both FPR1 expression (7.24±3.2 vs. 4.11±1.67 MFI, p<0.001) and FPR1/FPR2 MFI expression ratio (0.78±0.38 vs. 0.43±0.19, p = 0.001) on neutrophil were significantly increased in treatment-naïve OSA patients as compared with that in PS group. Sleep disordered breathing patients with hypertension had significantly decreased FPR2 expression on neutrophil (8.31±2.05 MFI, n = 18) as compared with those without hypertension (10.55±3.56, n = 44, adjusted p = 0.018, **S2A Fig**). Moreover, FPR3 expression of NK cell was further decreased in sleep disordered breathing patients with hypertension (2.06±0.41 MFI) as compared with that in those without hypertension (2.32±0.42 MFI, adjusted p = 0.009, **S2B Fig**).

### Increased FPR1/FPR2 expression ratio on neutrophil in sleep disordered breathing patients with excessive daytime sleepiness

Sleep disordered breathing patients with excessive daytime sleepiness had significantly increased FPR1/FPR2 expression ratio on neutrophil (0.86±0.47, n = 23) as compared with those without excessive daytime sleepiness (0.62±0.28, n = 39, adjusted p = 0.041, **S2C Fig**).

### Decreased FPR1 expression and FPR1/FPR2 expression ratio on CD16^+^ neutrophil, and increased FPR3 expression of CD3^-^CD56^+^NK cell after 6-month CPAP treatment

**Fig 4:**
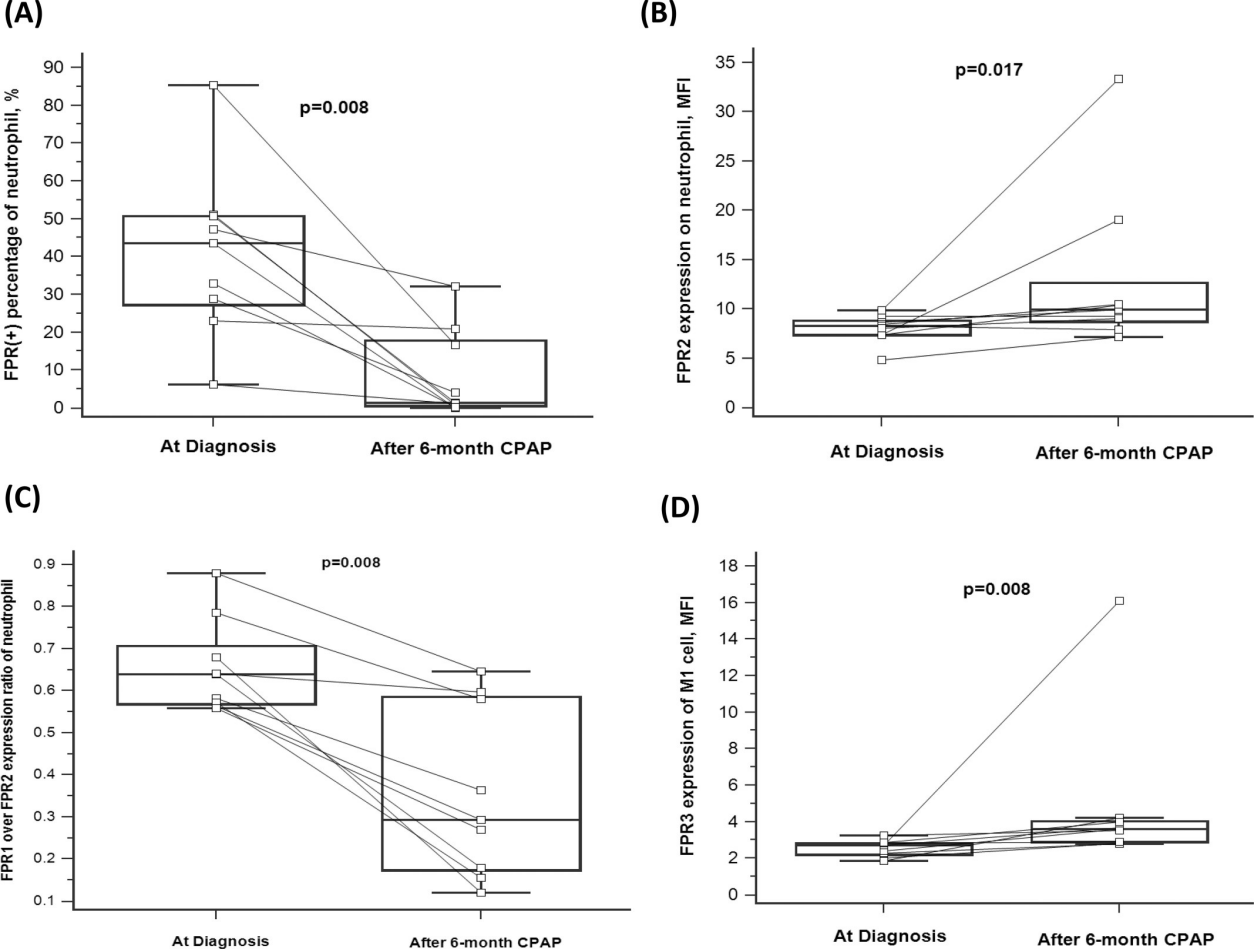
FPR1/2/3 expression changes of blood immune cells after 6-month CPAP treatment. (A) FPR1 (+) percentage on neutrophil was decreased after > 6-month CPAP treatment. (B) FPR2 expression on neutrophil was increased after >6-month CPAP treatment. (C) FPR1/FPR2 expression ratio on neutrophil was decreased after > 6-month CPAP treatment. (D) FPR3 expression of M1 cell was increased after > 6-month CPAP treatment. The box plots show the 25th, 50th, 75th percentiles, maximum, and minimum.

To determine the long-term effect of CPAP treatment on FPR expressions, we compared the flowcytometry data before and after more than 6-month CPAP treatment at home in 9 selected treatment-naïve OSA patients. Cell surface FPR1 expression (percentage of FPR1 positive cells in CD16^+^ neutrophil: 40.9±22.2% vs. 8.5±11.7, p = 0.008, **Fig 4A**) and FPR1/FPR2 MFI expression ratio (0.656±0.111 vs. 0.357±0.304, p = 0.008, **Fig 4B**) were both significantly decreased after > 6-month home CPAP treatment as compare with that at the first time PSG study, while FPR2 MFI expression on CD16^+^ neutrophil was increased (8±1.4 vs. 12.9±8.3, p = 0.017, **Fig 4C**). Furthermore, intracellular FPR3 expression of M1 cell was significantly increased after > 6-month CPAP treatment as compared with that at the first time PSG study (2.5±0.4 vs. 4.8±4.2 MFI, p = 0.008, **Fig 4D**).

### Impaired endothelial function in 6 selectedsevere OSA patients, and correlated with increased FPR2 expression on NK cell

Flow mediated dilatation percentage over right brachial artery, intima-media thickness and arterial stiffness of bilateral carotid arteries were assessed by ultrasound using the 7–12 MHz linear array transducer in 6 very severe OSA patients (AHI 68.4±13.2) and 6 HS (AHI 2.0±1.8). The very severe OSA patients had significantly decreased flow mediated dilatation percentage (2.3±1.06 vs. 7.47±1.08, p = 0.004, **S2D Fig**) but similar intima-media thickness (0.6±0.2 vs. 0.5±1.3 mm, p = 0.512, **S2E Fig** and arterial stiffness (8.9±2.7 vs. 9.1±2.6, p = 0.937, **S2F Fig**) as compared with the HS. FPR2 expression on NK cell was significantly increased in the very severe OSA patients versus the HS (12.94±1.75 vs. 9.13±1.98 MFI, p = 0.01, **S2G Fig**), and was negatively correlated with flow mediated dilatation percentage (r = -0.647, p = 0.012, **S2H Fig**).

### Defective LXA4 and RvD1 production in patients with sleep-disordered breathing

**Fig 5:**
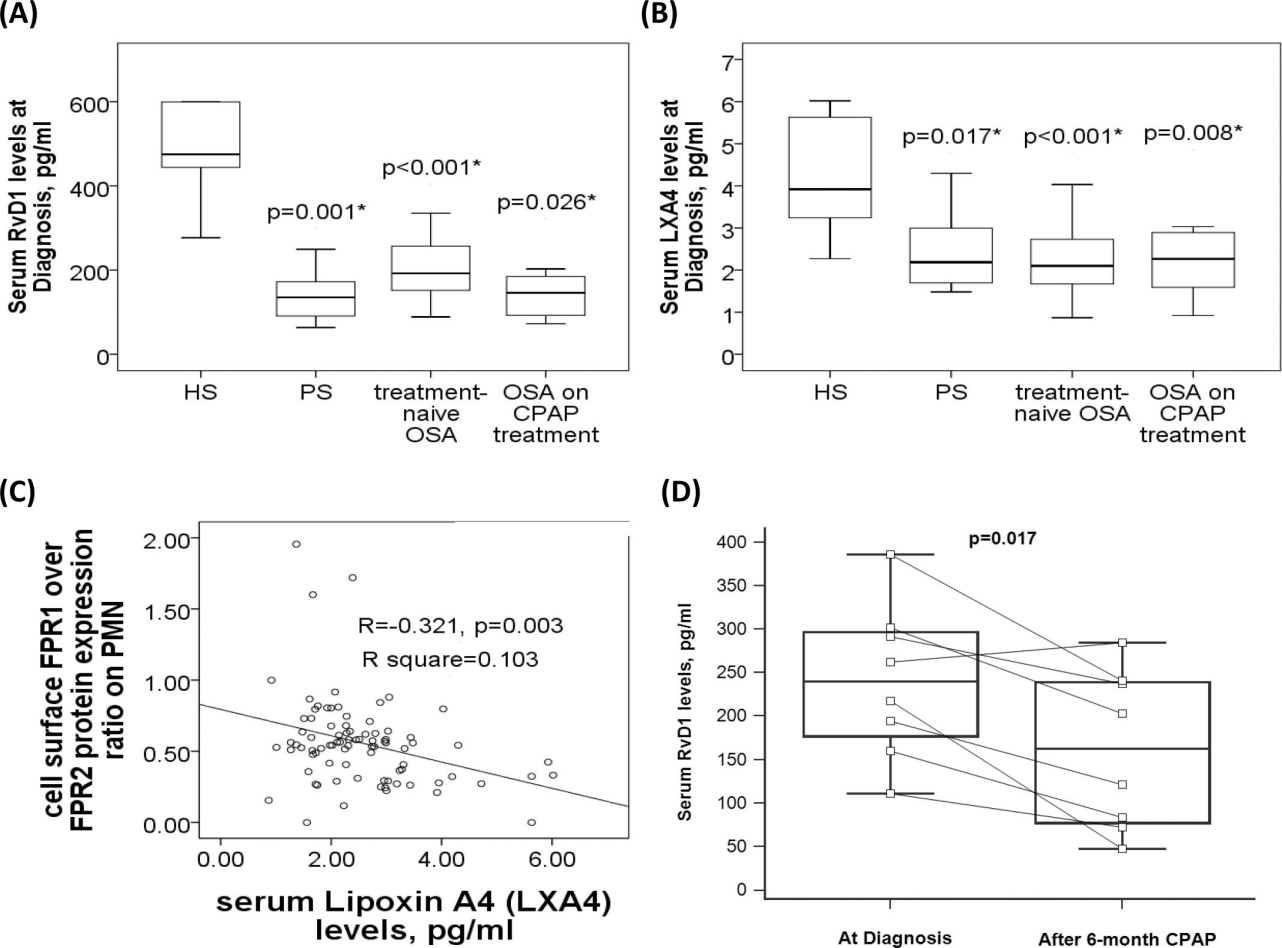
Serum lipoxin A4 (LXA4) and resolvin D1 (RvD1) deficiency in patients with sleep-disordered breathing. (A) Serum RvD1 and (B) LXA4 levels were both decreased in patients with primary snoring (PS), treatment-naïve OSA, and severe OSA on CPAP treatment as compared with that in the healthy subjects (HS). (C) Serum LXA4 was negatively correlated with cell surface FPR1/FPR2 expression ratio on blood neutrophil. (D) Serum RvD1 levels were further reduced after > 6-month CPAP treatment in 9 selected OSA patients. The box plots show the 25th, 50th, 75th percentiles, maximum, and minimum. *P<0.05 for comparisons between HS and another groups by Kruskal-Wallis test followed by post-hoc corrections.

There were significant between-group differences in serum LXA4 and RvD1 (both p values <0.001). Post hoc comparisons with corrections for multiple comparisons and adjustment for confounding factors revealed that serum Serum RvD1 levels were significantly decreased in patients with PS (141.6±55.8, p<0.001/adjusted p = 0.001), treatment-naive OSA (201.9±79.3, p<0.001/adjusted p<0.001), and severe OSA on CPAP groups (168±102.1 pg/ml, p<0.001/adjusted p = 0.026) as compared with that in HS (564±247.9 pg/ml, **Fig 5A**). Likewise, LXA4 levels were significantly decreased in patients with PS (2.46±0.88, p<0.001/adjusted p = 0.01), treatment-naive OSA (2.21±0.7, p<0.001/adjusted p<0.001), and severe OSA on CPAP groups (2.17±0.69 pg/ml, p<0.001/adjusted p = 0.008) as compared with that in HS (4.19±1.27 pg/ml, **Fig 5B**). Furthermore, serum LXA4 levels were negatively correlated with cell surface FPR1 over FPR2 protein expression ratio on neutrophil (**Fig 5C**). There was no significant difference in ANXA1, SAA, or LL-37 levels among the four groups. In the 9 OSA patients who received home CPAP treatment, serum RvD1 levels were further reduced 6 months later (161.3±90.8 versus 240.6±87.9 pg/ml, p = 0.017, **Fig 5D**), while no significant change was found for the other 4 FPR ligands. **Table 2** summaries the positive results of the expressions of the three FPRs and five FPR ligands in the current study.

**Table 2:** Summary of the positive results of the protein expressions of the three FPRs and five FPR ligands in the current study.

Biomarkers	Up- or down-regulation in OSA patients	Up- or down-regulation in clinical phenotypes	Longitudinal changes after CPAP treatment
FPR1 expression on neutrophil	Up-regulated	nil	Reduced
FPR2 expression on neutrophil	Down-regulated	Down-regulation with hypertension	Elevated
FPR1/FPR2 expression ratio on neutrophil	Increased	Further increased with excessive daytime sleepiness	Decreased
FPR3 expression of M1 monocyte	Down-regulated	nil	Elevated
FPR3 expression of M2a monocyte	Down-regulated	nil	nil
FPR3 expression of NK cell	Down-regulated	Down-regulation with hypertension	nil
Serum RvD1	Down-regulated	nil	Reduced
Serum LXA4	Down-regulated	nil	nil

### Effects of short-term IHR on FPR1/2/3 expressions of human monoccyte cell lines

**Fig 6:**
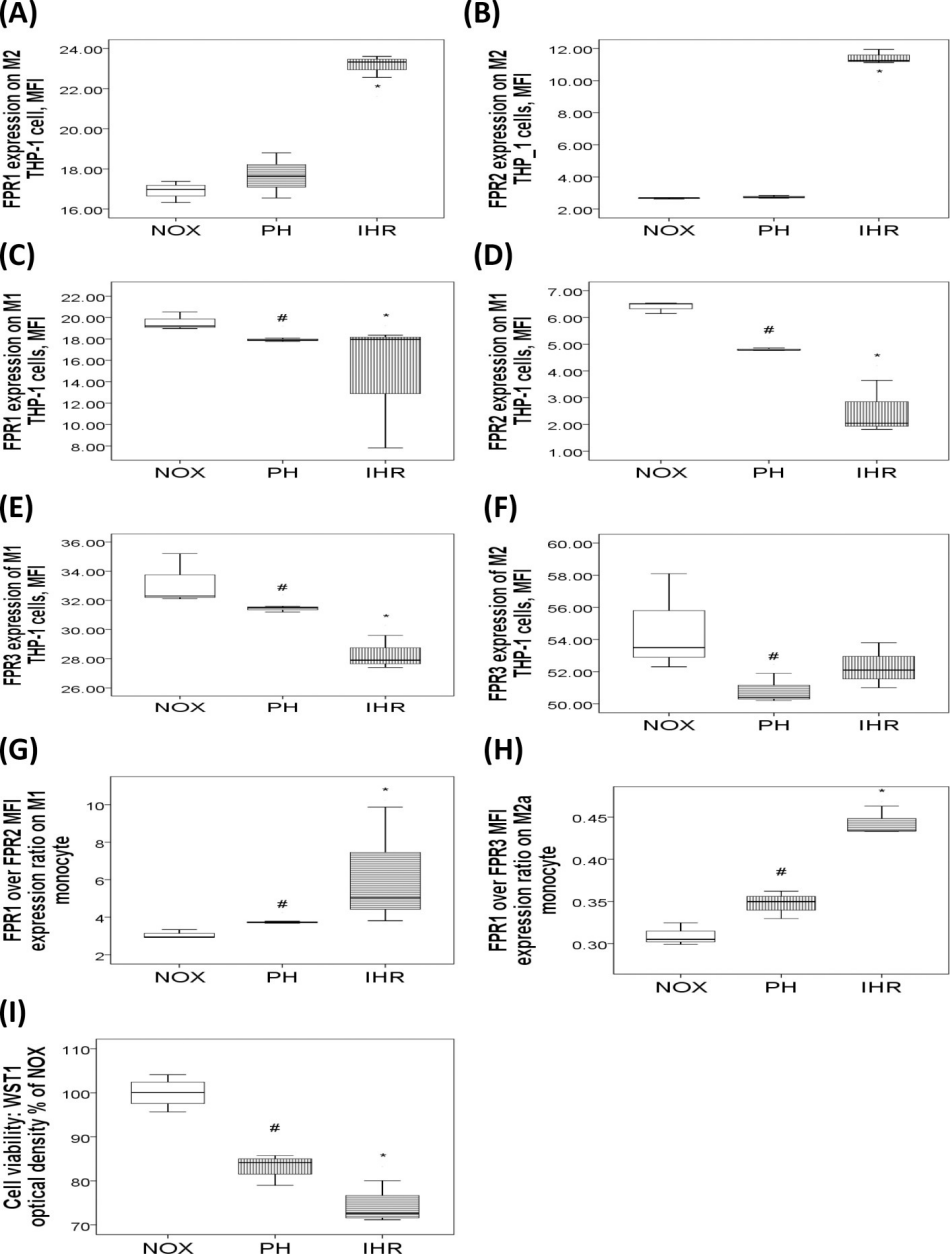
FPR1/2/3 expressions of human monocytic THP-1 cells exposed to normoxia (NOX), 2 days of persistent hypoxia (PH), or 2 days of intermittent hypoxia with re-oxygenation (IHR). The box plots show the 25th, 50th, 75th percentiles, maximum, and minimum. IHR treatment resulted in increased (A) FPR1 and (B) FPR2 expressions on M2a cells. Both IHR and PH treatment resulted in decreased (C) FPR1, (D) FPR2, and (E) FPR3 expressions on CD14^+^CD209^-^M1 cells. PH and IHR treatment resulted in decreased (F) FPR3 expression of CD14^+^Cd209^+^M2a cells. Both PH and IHR (G) increased FPR1/FPR2 expression ratio of M1 cell, and (H) increased FPR1/FPR3 expression ratio of M2a cell. (I) Cell viability was decreased in both PH and IHR versus NOX condition. *p<0.05 for comparisons between IHR and NOX conditions by U test #p<0.05 for comparisons between PH and NOX conditions by U test.

To determine whether IHR per se can affect FPR1/2/3 expressions, THP-1 cells were treated with 7 cycles of IHR/day for 2 days, 7 hours of PH/day for 2 days, or normoxic condition. IHR but not PH significantly increased FPR1 and FPR2 expressions on CD14^+^CD209^+^M2a cells in terms of MFI as compared with NOX condition (both p values<0.05, **Fig 6A** and **6B**), while both IHR and PH significantly decreased FPR1/2/3 expressions of CD14^+^CD209^-^M1 cells (all p values<0.05, **Fig 6C**, **6D** and **6E**). Both PH and IHR significantly decreased FPR3 expression of M2a cell (p<0.05, **Fig 6F**). Additionally, both PH and IHR increased FPR1/FPR2 expression ratio of M1 cell (p values<0.05, Fig 6G), and increased FPR1/FPR3 expression ratio of M2a cell (p values<0.05, Fig 6H). Cell viability, as measured by WST1 optical density, was decreased in both PH and IHR versus NOX condition, and further decreased in IHR versus PH (all p values<0.05, Fig 6I).

## Discussion

Prolonged neutrophil survival and increased superoxide release along with persisted low-grade systemic inflammation are evident in patients with OSA characterized by chronic IHR injury [24–26]. Increased neutrophil-to-lymphocyte ratios are associated with the development of endothelial dysfunction, leading to atherosclerosis and hypertension ultimately[27]. Recent studies showed that FPR1 signaling induces a rapid, but reversible, increase in the mitochondrial membrane potential within neutrophils with an associated oxidative burst and coordinated migratory activity, while FPR2 homodimerization inhibits neutrophil migration and concomitantly augments monocyte recruitment[28, 29]. In this study, we sought to investigate OSA-associated FPR1 and FPR2 expressions on innate immune cells, including neutrophil, monocyte and NK cell. We have shown, for the first time, the relationship between the altered FPR1/2 expression on neutrophil and the development of OSA. Furthermore, we found increased FPR1/FPR2 ratio and insufficiency of FPR2 and FPR3 in the OSA patients with EDS and hypertension phenotypes, respectively, while long-term CPAP treatment decreased FPR1 expression and FPR1/FPR2 expression ratio, and increased FPR3 and FPR2 expression. Additionally, in vitro IHR resulted in FPR1/2 up-regulation of anti-inflammatory M2a cells, and FPR1/2/3 down-regulation of pro-inflammatory M1 cells.

Previous studies have shown the importance and functional relevance of FPR1 in the pathogenesis of a range of both sterile and infective inflammatory conditions, suggesting that FPR1 is a key positive regulator of the inflammatory environment[30–32]. In the present study, both FPR1 expression and FPR1/FPR2 ratio on neutrophil were increased in OSA patients, and the latter further increased in those with excessive daytime sleepiness. The reversion of both FPR1 and FPR1/FPR2 ratio after > 6-month home CPAP treatment indicate that chronic IHR may play a role in FPR1 over-expression in treatment-naïve OSA patients. FPR 1 over-expression has been shown to be associated with tumor progression and survival in gastric cancer, indicating a link between FPR1-mediated pro-inflammatory cascades and poor outcome in cancer patients[33]. We speculate that chronic IHR-induced FPR1 over-expression on neutrophil may lead to pro-inflammatory responses and subsequent pronounced hypersomnia in OSA. Further study is required to clarify the cause and effect relationship between FPR1 over-expression and the development of the excessive daytime sleepiness phenotype in OSA.

Defective LPX A4 generation and FPR2 insufficiency has been demonstrated to reduce the ability of inhaled corticosteroids to impair control of airway inflammation in children with severe asthma[34]. A recent murine model of stroke showed that FPR 2 agonist could reduce leukocyte-endothelial interactions and initiate endogenous pro-resolving, anti-inflammatory pathways after cerebral ischemia/reperfusion injury [16]. A recent study demonstrates that endothelial progenitor cell homing was promoted through FPR2 signaling[35]. In the present study, we found that FPR2 expression on neutrophil was decreased in OSA patients and further decreased in those with hypertension. The reversion of FPR2 under-expression in the long-term (> 1 year) severe OSA patients on CPAP as compared with that in the treatment-naïve OSA group but not in those after > 6-month CPAP home treatment in the 9 selected patients indicate that FPR2 insufficiency induced by chronic IHR in OSA may require a longer time to recovery. On the other hand, paradoxical increase in FPR2 expression on NK cell was noted in a small subset of severe OSA patients, and correlated with endothelial dysfunction. FPR2 agonist has been shown to promote NK cell migration through ERK activation, and to provides cardiac protection through mobilization of circulating angiogenic cells after myocardial infarction in murine models[15, 36, 37].

Moreover, NK-cells play an essential role in angiotensin II-induced vascular dysfunction in a murine model[38]. Thus, we speculate that chronic IHR may trigger NK cell migration through FPR2 activation, leading to endothelial dysfunction in patients with severe OSA. Further study is required to clarify the role of FPR2 of NK cell in the protection from or contribution to endothelial dysfunction in OSA.

The physiological function of FPR3 is largely unknown, and little evidence for its relation with disease can be found. In the present study, we found that FPR3 expressions of both M1 monocyte and NK cell were decreased in OSA patients and the latter was further decreased in those with hypertension. Furthermore, FPR3 under-expression of NK cell was reversed after >6-month home CPAP treatment. In line with our findings, strain-specific loss of FPR 3 has been found in the murine vomeronasal and immune systems[39]. We speculate that human genetic variants of FPR3 and its under-expression may contribute to the development of the hypertension phenotype under chronic IHR stimuli in OSA syndrome. Further investigation is required to reveal underlying mechanisms by which insufficient FPR2 and FPR3 in OSA leads to the development of its adverse consequences.

It has been demonstrated that LXA4 reduces brain inflammation in subarachnoid hemorrhage rats and attenuates intestinal ischemia reperfusion injury through FPR 2/p38 MAPK and Keap1/Nrf2 signaling pathways, respectively[40–43]. Rv D1 has been shown to prime the resolution process initiated by calorie restriction in obesity-induced steatohepatitis through FPR2[17]. For the first time, we found defective LXA4 and RvD1 production in association with increased FPR1/FPR2 expression ratio on blood neutrophils in patients with sleep disordered breathing. The further reduction in the RvD1 levels after 6-month CPAP treatment may indicate that the defective RvD1 production could be caused by certain genetic variants rather than induced by chronic IHR. Hopefully, our results will lead to the development of novel therapeutic options of using synthetic LXA4/RvD1 peptides for OSA in cases where an optimal anti-oxidant and anti-inflammatory treatment modality is needed but still lacking.

The limitations of this study should be acknowledged. First, categorizing those without habitual snoring in the absence of polysomnography results into HS is the major limitation of this study, because patients with central sleep apnea may have intermittent hypoxemia without snoring and people who are unaware of snoring may falsely reported as non-snorer in the absence of observations from bed partners[44]. However, each bed partner of the HS reported no observation of snoring during the sleep, indicating a low possibility that these subjects would have OSA. Central sleep apnea seems to be less prevalent in Taiwan according to our experiment, although no publications of the epidemiology studies in Asian could be found. It occurs mainly in patients with heart failure, while no subject in the HS group had a history of heart failure. Second, many confounding factors may affect FPR 1/2/3 expressions in the cross-sectional comparisons of the four study groups. However, the four groups were matched in terms of age, percentage of co-morbidity, smoking history, and biochemical profiles. Moreover, longitudinal follow-up in a subset of OSA patients receiving CPAP treatment showed partial reversion of the altered FPR expressions. Third, in the subgroup analysis, the two compared groups were not matched in terms of certain co-variables. However, we made multiple linear regression analyses to minimize the effect of confounding factors and get adjusted p values. Fourth, the sample size in the subgroup analysis of the correlation between flow mediated dilatation and FPR expressions was relatively small in addition to the existence of outliers. Further investigation with gain or loss of function method is required to establish the cause and effect relationship between endothelial dysfunction and aberrant FPR1/2/3 expressions. Finally, the present data were insufficient to prove a causative role of FPRs in the development of OSA. However, the preliminary in vitro short-term IHR experiment showed that FPR 1/2/3 could be differentially expressed in different subpopulations of human monocytic cells. Specifically, both FPR1/FPR2 expression ratio of M1 cell and FPR1/FPR3 expression ratio of M2a cell were increased in response to PH and IHR stimuli, indicating that hypoxia might up-regulate pro-inflammatory FPR1 expression and down-regulate anti-inflammatory FPR2/3 expression. These findings were in accordance with the results of the human immune cells, which showed up-regulation of FPR1/FPR2 expression ratios in the neutrophils from treatment-naïve OSA patients. Thus, our findings open the possibility of using synthetic FPR1 antagonists and FPR2/FPR3 agonists to resolve persistent inflammation and subsequent endothelial dysfunction in OSA[45]. Further investigations on the effects of FPR ligands on immune cells under IHR stimuli, and epigenetic mechanisms by which IHR leads to altered FPR signaling are ongoing.

## Conclusions

We have found a link of FPR1 over-expression and FPR2 under-expression on blood neutrophil along with defective production of LXA4/RvD1, as well as FPR3 insufficiency of M1 monocyte, M2a monocyte, and NK cell, to OSA and its adverse consequences, including hypertension and EDS. CPAP treatment partly reversed the altered expressions of FPR1/2/3. FPR1 over-expression and FPR2/FPR3 insufficiency in association with under-production of specific FPR ligands may be associated with disease severity and adverse consequences of OSA.

## Supporting information

S1 Fig Flow cytometric analysis of formyl peptide receptor expressions of human innate immune cells. (A) Un-gated forward and side-scatter plot for blood innate immune cells, in which neutrophil is shown in pink, monocyte in green, and lymphocyte in red. (B) M1 monocyte was identified by CD14 positive and CD209 negative cells, while M2a monocyte by CD14 and CD209 double positive cells. Dual parameter plot of fluorescence of monocyte labeled with FPR1-CFS and FPR2-PE. (C) Neutrophil was identified by CD16 positive cells. Dual parameter plot of fluorescence of neutrophil labeled with FPR1-CFS and FPR2-PE. (D) NK cell was identified by CD3 negative and CD56 positive lymphocyte, while NK T cell by CD3 and CD56 double positive cells. Representative histograms of (E) cell surface FPR1 expression on neutrophil, and (F) cell surface FPR2 expression on neutrophil.
(TIF)

S2 Fig Differential FPR expressions of blood immune cells in OSA patients with various clinical phenotypes. (A) FPR2 expression on neutrophil was decreased in sleep disordered breathing patients with hypertension. (B) FPR3 expression of NK cell was decreased in sleep disordered breathing patients with hypertension. (C) FPR1/FPR2 expression ratio was increased in sleep disordered breathing patients with excessive daytime sleepiness. (D) Flow-mediated dilatation was decreased in a small subset of very severe OSA patients. (E) Intima media thickness and (F) stiffness of left common carotid artery were similar between the very severe OSA patients and HS. (G) FPR2 expression on NK cell was increased in a small subset of very severe OSA patients. (H) FPR2 expression on NK cell was negatively correlated with flow mediated dilatation.
(TIF)

## Abbreviations

OSA: Obstructive sleep apnea
IHR: intermittent hypoxia with re-oxygenation
AHI: apnea hypopnea index
CPAP: continuous positive airway pressure
LXA4: lipoxin A4
RvD1: resolvin D1
ANXA1: annexin A1
SAA: serum amyloid A
LL-37: Cathelicidin
PS: primary snoring
SaO2: oxyhemoglobin saturation
HS: healthy subjects
NK: Natural killer
MFI: mean fluorescence intensity
ESS: Epworth Sleepiness Scale
ODI: oxygen desaturation index
