# Supplementary figures and images for: Formyl peptide receptor 1 up-regulation and formyl peptide receptor 2/3 down-regulation of blood immune cells along with defective lipoxin A4/resolvin D1 production in obstructive sleep apnea patients
Source: PLoS One. 2019 May 22;14(5):e0216607. doi: 10.1371/journal.pone.0216607 (PMC6530856; doi:10.1371/journal.pone.0216607)

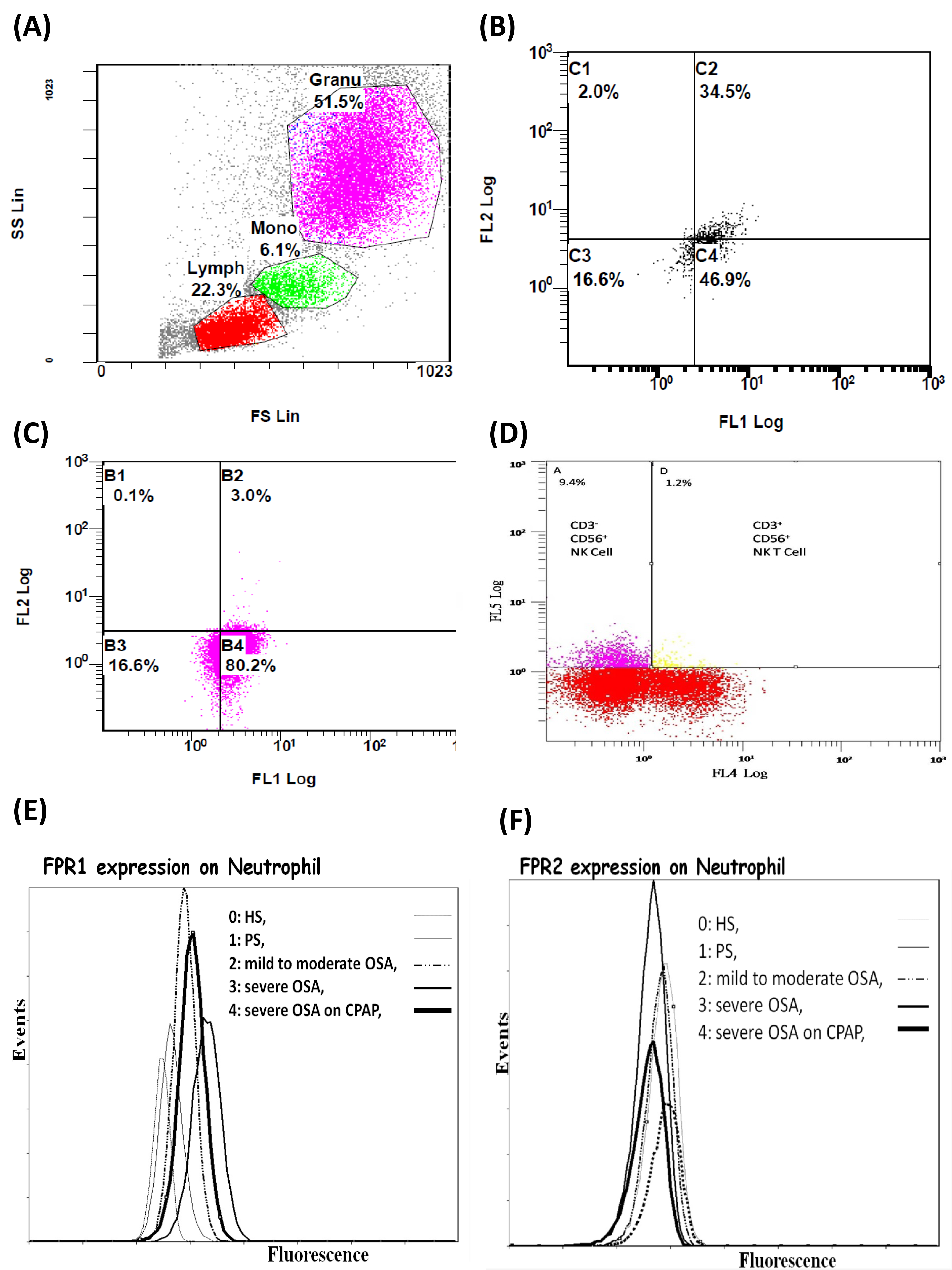

Supplement: S1 Fig — (A) Un-gated forward and side-scatter plot for blood innate immune cells, in which neutrophil is shown in pink, monocyte in green, and lymphocyte in red. (B) M1 monocyte was identified by CD14 positive and CD209 negative cells, while M2a monocyte by CD14 and CD209 double positive cells. Dual parameter plot of fluorescence of monocyte labeled with FPR1-CFS and FPR2-PE. (C) Neutrophil was identified by CD16 positive cells. Dual parameter plot of fluorescence of neutrophil labeled with FPR1-CFS and FPR2-PE. (D) NK cell was identified by CD3 negative and CD56 positive lymphocyte, while NK T cell by CD3 and CD56 double positive cells. Representative histograms of (E) cell surface FPR1 expression on neutrophil, and (F) cell surface FPR2 expression on neutrophil. (TIF) [file pone.0216607.s001.tif]

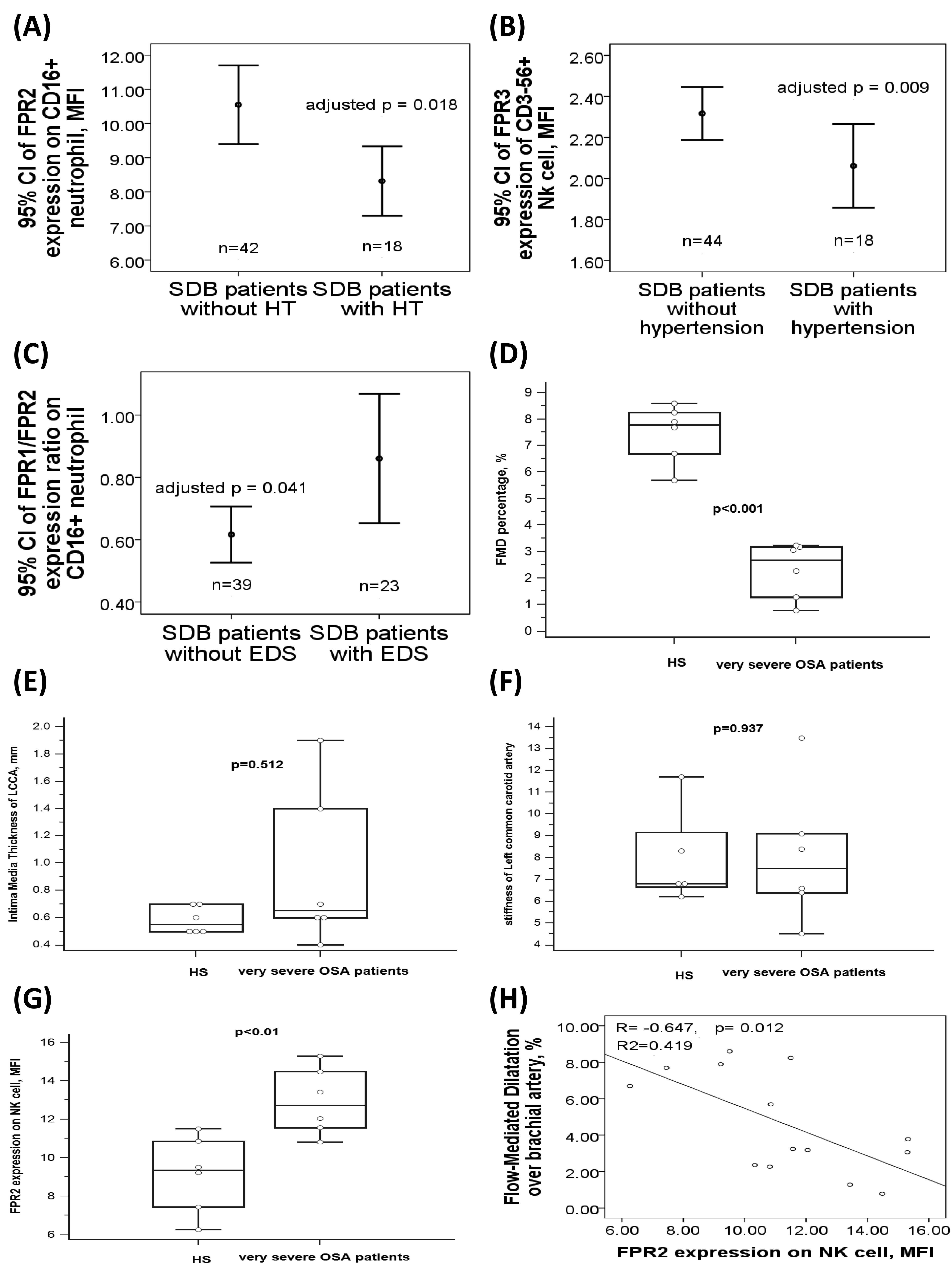

Supplement: S2 Fig — (A) FPR2 expression on neutrophil was decreased in sleep disordered breathing patients with hypertension. (B) FPR3 expression of NK cell was decreased in sleep disordered breathing patients with hypertension. (C) FPR1/FPR2 expression ratio was increased in sleep disordered breathing patients with excessive daytime sleepiness. (D) Flow-mediated dilatation was decreased in a small subset of very severe OSA patients. (E) Intima media thickness and (F) stiffness of left common carotid artery were similar between the very severe OSA patients and HS. (G) FPR2 expression on NK cell was increased in a small subset of very severe OSA patients. (H) FPR2 expression on NK cell was negatively correlated with flow mediated dilatation. (TIF) [file pone.0216607.s002.tif]
